# Achieving Luminescence of Sr_3_Ga_1.98_In_0.02_Ge_4_O_14_:0.03Cr^3+^ via [In^3+^] Substitution [Ga^3+^] and Its Application to NIR *pc*-LED in Non-Destructive Testing

**DOI:** 10.3390/molecules28248059

**Published:** 2023-12-13

**Authors:** Tao Wang, Bingkai Gao, Jiehong Li, Zhijun Wang, Panlai Li

**Affiliations:** 1 College of Science, China University of Petroleum (East China), Qingdao 266580, China; 2 Hebei Key Laboratory of Optic-Electronic Information and Materials, College of Physics Science & Technology, Hebei University, Baoding 071002, China

**Keywords:** Cr^3+^ doping, cation substitution, thermal stability, luminous intensity

## Abstract

Cr^3+^-doped Sr_3_Ga_2_Ge_4_O_14_:0.03Cr^3+^ (SGGO:0.03Cr^3+^) phosphor was synthesized via a high-temperature solid-phase method. Considering the tunable structure of SGGO, Ga^3+^ ions in the matrix were substituted with In^3+^ ions at a certain concentration. The tuned phosphor produced a red-shifted emission spectrum, with its luminescence intensity at 423 K maintained at 63% of that at room temperature; moreover, the internal quantum efficiency increased to 65.60%, and the external quantum efficiency correspondingly increased to 21.94%. On this basis, SGIGO:0.03Cr^3+^ was encapsulated into a *pc*-LED, which was applied in non-destructive testing (NDT) experiments, successfully realizing the recognition of water and anhydrous ethanol, proving its potential application in the field of NDT.

## 1. Introduction

In recent years, research on near-infrared light has continued to deepen due to its invisibility, high penetration, and non-destructive and other excellent characteristics; according to its specific band, near-infrared light is widely used in plant lighting, night vision lighting, non-destructive testing, medical testing, and other fields [1,2,3,4,5,6]. At present, halogen lamps and infrared lasers are the most common and representative traditional infrared light sources. However, halogen lamps have high power consumption, low efficiency, and short service life. Infrared lasers are large in size and high in cost, which limit their application in actual production work to a certain extent [7,8,9,10,11,12]. The emergence of near-infrared phosphor-converted light-emitting diodes (NIR *pc*-LEDs) has become a hot research topic due to their multiple advantages such as small size, energy savings, environmental protection, and simple preparation. which also increase the spectral coverage of LED devices over a larger area [13,14,15,16]. However, the emission wavelength coverage of LED chips is severely limited as their emission wavelength depends largely on the composition of the LED chip. To solve these problems, the industry has developed down-conversion phosphors, an important component of NIR *pc*-LEDs that affects the emission spectra of the light-emitting device, photoelectric efficiency, device lifetime, temperature characteristics, and many other critical aspects. For this reason, there is a need to develop high-performance NIR phosphors that are well matched to blue LED chips.

NIR phosphors are usually composed of activated ions and host compound components. Cr^3+^, Bi^3+^, Eu^2+^, and Mn^2+^ are typical activated ions that produce NIR phosphor emission peaks. Although the emission of Bi^3+^ and Eu^2+^ is led by the wavelength close to the deep red region, the half-peak width of NIR is still not wide. The application of Mn^2+^-doped oxide phosphors suffers from drawbacks such as a low color rendering index and high color temperature. These drawbacks limit their application in the NIR. To solve the above problems, Cr^3+^ has attracted much attention. It can simply modulate its emission by changing its local environment. The 3d^3^ electron leaps of Cr^3+^ are significantly affected by the crystal field. Depending on the strength of the crystal field environment, the typical luminescence of Cr^3+^ usually exhibits three features: spin-forbidden leaps ^2^E→^4^A_2_ are generated when Cr^3+^ is in a strong crystal field (*D_q_*/*B* > 2.3), and a narrow-band emission is produced near 700 nm. When Cr^3+^ is in a weak crystal field (*D_q_*/*B* < 2.3), a tunable broadband emission is generated by the spin-allowed jump ^4^T_2_→^4^A_2_; when in an intermediate crystal field (*D_q_*/*B* ≈ 2.3), the spectrum exhibits both of the above phenomena, Hence, the luminescence performance of Cr^3+^ can be flexibly tuned using these properties. It has been found that the cation substitution strategy is an effective method to improve the spectra and enhance the phosphor temperature performance [17,18,19,20,21,22,23]. This is because the change in cation components in the matrix material affects the overall crystal field environment, and Cr^3+^ is highly susceptible to the influence of the crystal field environment in which it is embedded. So, the luminescence performance of Cr^3+^ changes accordingly after the change in cation components in the matrix. Qiu et al. used Al^3+^ to replace Sc^3+^ on the host material Gd_3_Sc_2_Ga_3_O_12_ so that the internal quantum efficiency of the emission of Cr^3+^ was significantly increased to nearly 100%, and the temperature stability after modulation was improved to 86% after modulation [24]. Zhang et al. utilized [Lu^3+^-Al^3+^] to replace [Ca^2+^-Si^4+^] in Ca_2_LuZr_2_Al_3_O_12_:Cr^3+^, which broadened the spectrum and improved the emission intensity by changing the crystal field and electroacoustic coupling effects [11]. In previous experiments, through doping different concentrations of Cr^3+^ ions and comparing their luminescent properties, two excellent phosphors, Sr_3_Ga_2_Ge_4_O_14_:0.015Cr^3+^ and Sr_3_Ga_2_Ge_4_O_14_:0.03Cr^3+^, were obtained. Considering that the fluorescent materials with larger half-peak full-widths and higher temperature stability are more preferred in practical applications, we selected Sr_3_Ga_2_Ge_4_O_14_:0.03Cr^3+^ with a half-peak full width of 280 nm for further study [25].

Although some Cr^3+^-activated oxide phosphors [1,3,21,25,26,27,28,29,30,31] have been reported, no detailed report on the Cr^3+^-activated Sr_3_Ga_2_Ge_4_O_14_–Sr_3_In_2_Ge_4_O_14_ solid–solution system has been published to date. In this study, the NIR phosphor Sr_3_Ga_2−2*x*_In*_x_*Ge_4_O_14_:0.03Cr^3+^ (SGIGO:0.03Cr^3+^) with higher emission intensity and better temperature stability was obtained via In^3+^-Ga^3+^ cation substitution, and the luminescence performance of the phosphor was analyzed. Detailed characterization and analysis revealed that the enhancement in the material morphology led to a significant increase in the emission intensity of the spectrum, which was maintained at 63% of room temperature at 423 K. The spectral intensity of the fluorescent phosphor was found to be higher than that at room temperature. Finally, good application capability was demonstrated through liquid non-destructive identification experiments.

## 2. Results and Discussion

### 2.1. Phase Purity and Crystal Structure Analysis

**Figure 1:**
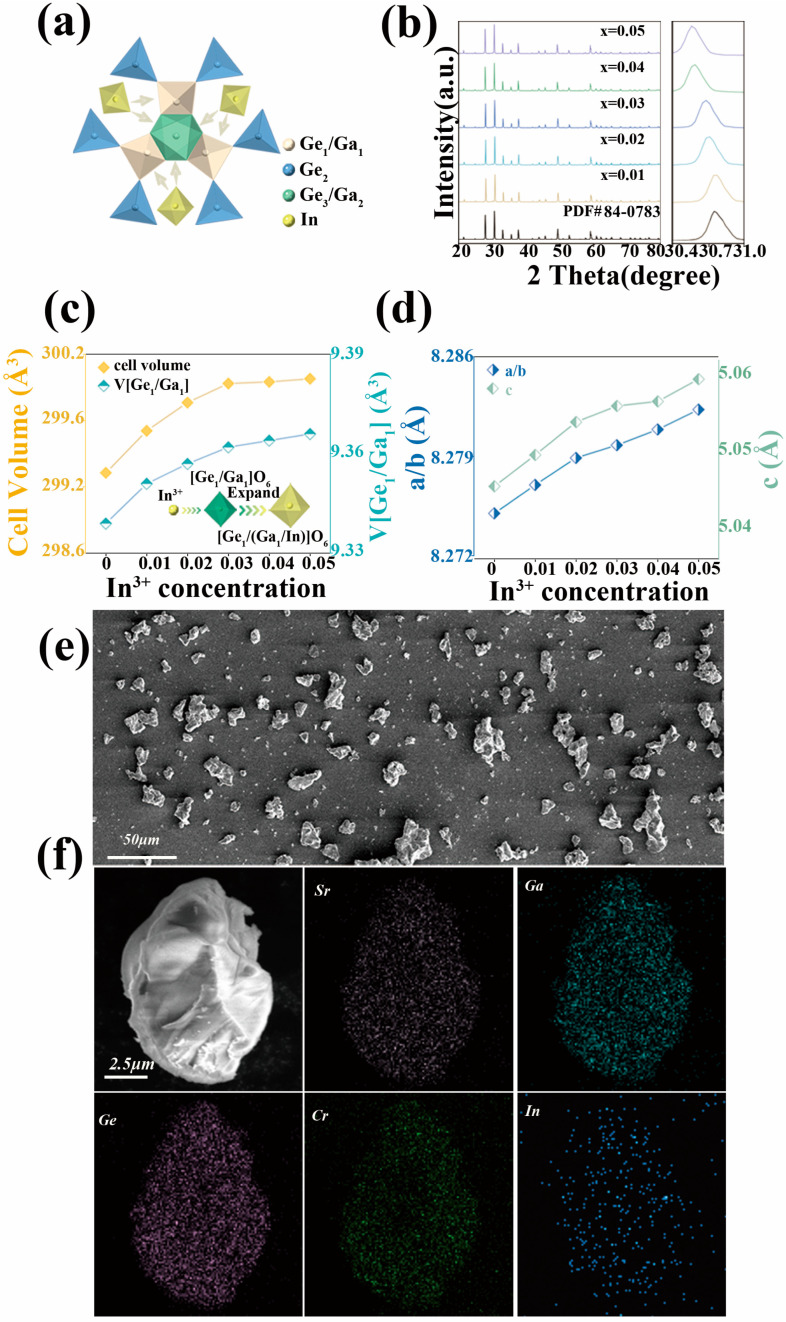
(**a**) Schematic structure of In^3+^ replacing Ga^3+^ in SGGO. (**b**) XRD patterns of SGIGO:0.03Cr^3+^ and standard data of SGGO (PDF# 84-0783). (**c**) SG_2−2*x*_I*_x_*GO:0.03Cr^3+^ (*x* = 0–0.05) cell volume V and [Ge_1_/Ga_1_]O_6_ volume variations. (**d**) Variations in cell parameters a/b and c. (**e**) SEM mapping of SG_1.98_I_0.02_GO:0.03Cr^3+^. (**f**) EDS mapping of SG_1.98_I_0.02_GO:0.03Cr^3+^.

For the cation modulation strategy, the theoretical value of the radius difference between the dopant ion and the substituted ion should be less than 30%, which is more promising. In Sr_3_Ga_2_Ge_4_O_14_, the radii of dopant ions and substituted ions are as follows: In^3+^ [CN = 6, R = 0.80].

(1) $$D_{r}=\left|100\times \frac{R_{m}(N)-R_{d}(N)}{R_{m}(N)}\right|$$

where *D_r_* is the radius difference between the doped and substituted ions; *R_m_* and *R_d_* denote the radii of the substituted and doped ions, respectively; and *N* denotes the coordination number. According to Equation (1), it can be calculated that *D_r_* [In^3+^-Ga^3+^] = 3%. It is obvious that the strategy of substituting In^3+^ ions for Ga^3+^ ions to modulate the crystal field is theoretically very feasible, and the substitution process is demonstrated in Figure 1a.

A series of SG_2−2*x*_I*_x_*GO:0.03Cr^3+^ (*x* = 0–0.05) samples were synthesized in the experiment using high temperature solid-phase method and their XRD diffraction data were tested as exhibited in Figure 1b. The XRD data of the samples did not exhibit any impurity peaks when compared with the SGGO standard card PDF#84-0783 (MDI Jade, version 1251), which indicates that the synthesized samples were all pure phases, and the introduction of In^3+^ ions did not affect the original structure of the material. In addition, when the crystalline phase was transformed from SGGO:0.03Cr^3+^ to SG_2−2*x*_I*_x_*GO:0.03Cr^3+^, the main peaks produced a shift towards a small angle, which can be explained by the Bragg equation [2]:

2*d* sin *θ* = *kλ* (2)

where *d* represents the intergranular spacing, *θ* represents the diffraction angle, *k* is the reflection level for a fixed value, and *λ* is the wavelength of the X-rays. From the formula, it can be seen that the intergranular spacing *d* is inversely proportional to the diffraction angle *θ*, which means that the larger the intergranular spacing, the smaller the diffraction angle. Due to the larger radius of In^3+^ ions, the process of replacing Ga^3+^ by In^3+^ leads to lattice expansion, and the intergranular spacing *d* then becomes larger, so the diffraction angle *θ* becomes smaller, which explains why the diffraction peaks in XRD are shifted to a small angle. In order to obtain more accurate crystal structure information, Rietveld refinement was performed, and the calculation results were obtained by the GSAS program. This is exhibited in Figure S1a,b (see Supplementary Materials). The Rietveld refinement results for SGGO:0.03Cr^3+^ and SG_1.98_I_0.02_GO:0.03Cr^3+^ were within reasonable limits, proving the good crystallinity of the samples. The Rietveld refinement results for the remaining samples are exhibited in Figure S1c–f (see Supplementary Materials).

As seen from the Rietveld refinement results, the cell volume V and the volume of [Ge_1_/Ga_1_] O_6_ kept increasing with the increase in the doping concentration of the In^3+^ ions. The lattice parameters a/b and c exhibited the same trend, as shown in Figure 1c,d. This is due to the fact that the radius of In^3+^ ions is larger than that of Ga^3+^ and, when doped, the lattice expands as a result.

In order to further analyze the morphology and elemental distribution of the synthesized samples, SEM tests were carried out on the samples, as exhibited in Figure 1e. The SEM images show that the material is a particle with a diameter of about 10 μm. In Figure 1f, it can be seen that the elemental distribution is uniform, and the above results demonstrate the successful doping of In^3+^.

### 2.2. Luminescence Characterization and Mechanism of Sr_3_Ga_1.98_In_0.02_Ge_4_O_14_:0.03Cr^3+^

**Figure 2:**
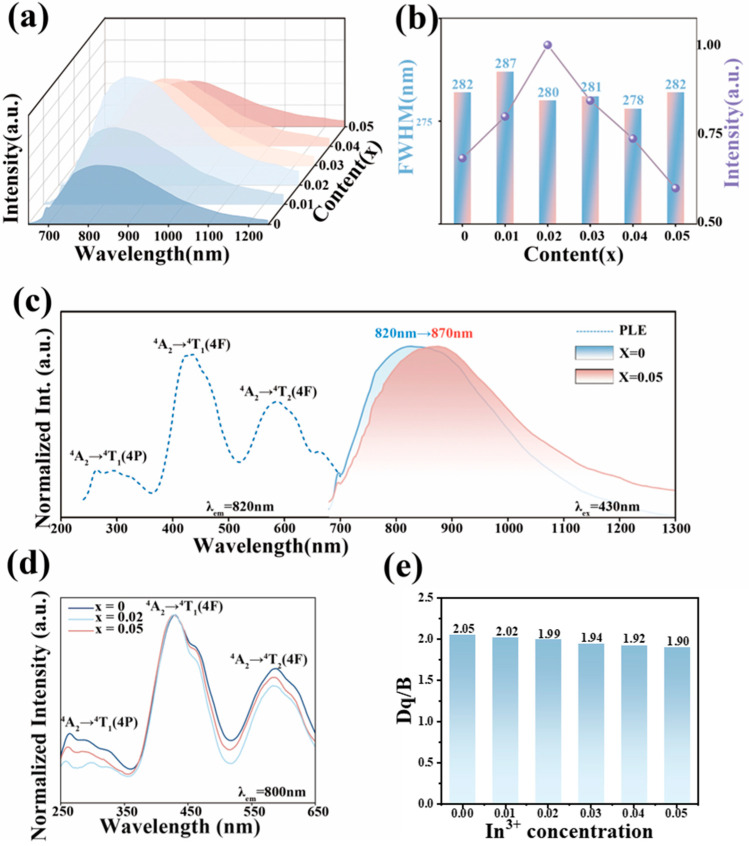
(**a**) Emission spectra of SG_2−2*x*_I*_x_*GO:0.03Cr^3+^ (*x* = 0–0.05); (**b**) FWHM and emission intensity of SG_2−2*x*_I*_x_*GO:0.03Cr^3+^ (*x* = 0–0.05); (**c**) excitation and emission spectra of SG_1.95_I_0.05_GO:0.03Cr^3+^, and SGGO:0.03Cr^3+^ emission spectra of SGGO; (**d**) excitation spectra of SG_2−2*x*_I*_x_*GO:0.03Cr^3+^ (*x* = 0–0.05); (**e**) crystal field intensities *D_q_*/*B* of SG_2−2*x*_I*_x_*GO:0.03Cr^3+^ (*x* = 0–0.05).

Figure 2a exhibits the emission spectra of SG_2−2*x*_I*_x_*GO:0.03Cr^3+^ (*x* = 0–0.05) monitored at room temperature, which highlights that a trend in the luminescence intensity of increasing and then decreasing with the increase in the In^3+^ ion concentration. The intensity reaches the maximum value at 2% In^3+^ ion concentration, which is about 1.84 times of the luminescence intensity when undoped with In^3+^. Throughout the substitution process, the half-peak width of the emission spectra was maintained at about 280 nm, as shown in Figure 2b. Figure 2c exhibits the normalized excitation and emission spectra, and it can be seen that the shape of the excitation spectra after In^3+^ ion doping did not exhibit any significant changes and still agreed with the typical excitation spectral shape of Cr^3+^. In addition, the spectra produced a red shift of 50 nm with the increase in the In^3+^ concentration, which was related to the electron cloud expansion effect and the change in the crystal field strength. It can be understood like this: The mass number of In is larger than that of Ga. Adding the atom with the larger mass number ensures a “larger lattice constant”. Thus, the electron orbital clouds in the cation atoms enable shielding or screening of the crystal-field strength, resulting in a smaller value of *D_q_*. Therefore, it ensures a smaller emission energy of ^4^T_2_ (i.e., the red shift in the emission wavelength) [4,21,32]. Previous research by solid-state scientists showed that when *x* changes from 0 to 0.05, it does not cause a significant change in the band-gap energy [33].

The electron cloud extension effect refers to the phenomenon that the more covalent an ion, the more diffuse its electron orbitals. The interactions between electrons are reduced, resulting in a shift in the jump energy between the electron energy levels in the lower energy direction. This is usually expressed using the following equation [34]:

1 − *β* = *hk* (3)

where *β* denotes the electron cloud expansion effect, and *h* and *k* are the electron cloud expansion factors, which are related to the elemental electronegativity. The smaller the electronegativity of an element, the stronger the covalency, and the more pronounced the electron cloud expansion effect of the compound. Its spectral line shifts to the long-wave direction [27,35,36,37,38]. For Ga^3+^ and In^3+^, whose electronegativity is 1.81 and 1.78, respectively, when In^3+^ replaces Ga^3+^, the electron cloud extension effect is enhanced, resulting in a red shift in the emission spectra of SG_2−2*x*_I*_x_*GO:0.03Cr^3+^.

Figure 2d exhibits the excitation spectrum of SG_2−2*x*_I*_x_*GO:0.03Cr^3+^ (*x* = 0–0.05) monitored at 800 nm, which was normalized in order to see the change in the excitation spectrum after In^3+^ doping more clearly. The three excitation bands belong to the typical ^4^A_2_→^4^T_1_(^4^P), ^4^A_2_→^4^T_1_(^4^F), and ^4^A_2_→^4^T_2_(^4^F) leaps of Cr^3+^, respectively. It can be seen that, with the increase in the doping concentration of In^3+^, the peak position of ^4^A_2_→^4^T_1_(^4^F) is basically unchanged, whereas that of ^4^A_2_→^4^T_2_(^4^F) is more affected and shifted. In the previous section, we mentioned that the broadband emission of SGGO:Cr^3+^ under optimal doping is attributed to the radiative jump from the excited state ^4^T_2_(F) to the ground state ^4^A_2_(F). When exactly determining the various excited-state energies from experimental optical spectra for calculating Equations (4)–(6), one must consider the strong phonon coupling nature of the 3d^3^ (Cr^3+^) electrons with the phosphor host lattices [39,40]. Such excited-state energies to be used in the Racah parameter calculations in Equations (4)–(6) cannot be simply obtained from the peak energy of each PL or PLE band. It has been demonstrated that the use of such emission or excitation “peak” energies, but not the “zero-phonon line (ZPL)” energies, in calculating Racah parameters (such as *D_q_*, *B*, and *C*) usually results in unacceptable conclusions [41,42]. For simplicity, however, we considered the “peak” energy of the various excited states taken from the experimental optical spectra for calculating Equations (4)–(6) with a suitable assumption of a Racah parameter ratio of *C*/*B* [41]. This approach is the same as those reported in the previous literature, but leaves problems to be solved in the near future [42].

(4) Dq=E(T24)=E(A24→T24)−ΔS/2

(5) $$\frac{D_{q}}{B}=\frac{15(\Delta E/D_{q}-8)}{{(\Delta E/D_{q})}^{2}-10(\Delta E/D_{q})}$$

(6) ΔE=E(T14)−E(T24)=E(A24→T14)−E(A24→T24)

### 2.3. Temperature Stability and Quantum Efficiency Analysis of Sr_3_Ga_1.98_In_0.02_Ge_4_O_14_:0.03Cr^3+^

The variation of luminescence intensity with temperature may be affected by a variety of factors, such as lattice vibrations, changes in energy level transitions, and changes in material structure. Some luminescent materials undergo structural phase transitions or diffusion of lattice defects at high temperatures, leading to a decrease in luminescence intensity. In a previous study of Rb_2_GeF_6_:Mn^4+^, it was found that the luminescence intensity was enhanced with increasing temperature in the range of 420 K to 450 K, and gradually weakened as the temperature increased at T > 450 K, which was the cause of thermal burst [43]. Therefore, temperature stability is an important factor to determine whether the luminescent material can realize successful application. For this purpose, the variable temperature spectrum of SG_1.98_I_0.02_GO:0.03Cr^3+^ was tested, as shown in Figure S2 (see Supplementary Materials), and its luminescence intensity was maintained at 63% of that at room temperature at 423 K, which is a significant enhancement compared to that of the unmodulated SGGO:0.03Cr^3+^ (56%) in the previous chapter.

**Figure 3:**
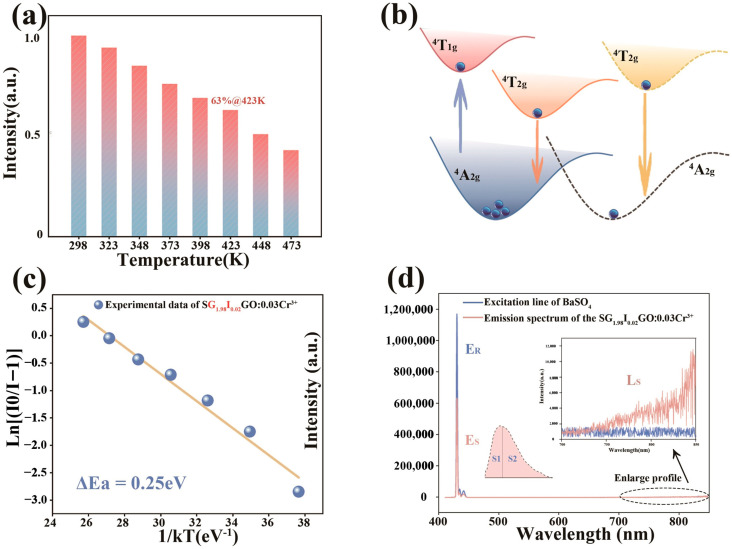
(**a**) Variation in emission intensity with temperature for SG_1.98_I_0.02_GO:0.03Cr^3+^; (**b**) positional coordinate plot of SG_1.98_I_0.02_GO:0.03Cr^3+^; (**c**) activation energy plot of SG_1.98_I_0.02_GO:0.03Cr^3+^ using the Arrhenius equation; (**d**) internal quantum efficiency of SG_1.98_I_0.02_GO:0.03Cr^3+^, where the inset exhibits a schematic diagram of the tested and fitted ranges.

In general, a more backward emission position, a larger half-peak width, and a higher temperature stability often do not go hand in hand because a stronger electroacoustic coupling effect leads to a stronger temperature burst phenomenon [44]. The reason for the elevated temperature stability after modulation can be explained by the bit pattern coordinate plot as shown in Figure 3b.

There are generally two ways for electrons to return to the ground state from the excited state, one of which is through radiative leaps [45]. In the previous section through calculations, we know that the doping of In^3+^ ions reduces the crystal field strength, which can be seen in the T-S diagrams, where the position of the ^4^T_2_ energy level should correspondingly be lowered, as exhibited in Figure 3b, and therefore the energy radiated in the process of returning to the ground state becomes smaller; this also explains the spectral red shift.

The other way is that the electron reaches the intersection of the ground and excited states through lattice relaxation and returns to the ground state via radiation with fewer leaps [46,47]. The energy required in this process is the activation energy Ea. The activation energy of SG_1.98_I_0.02_GO:0.03Cr^3+^ can be seen in the slope in Figure 3c. It was calculated using Equation (7) to be 0.25 eV, which is increased by 0.03 eV compared to the undoped state. Therefore, the doping of In^3+^ increases the activation energy of the material and decreases the chances of electron-radiation-free leaps, which leads to an improvement in the temperature stability of the material.

(7) $$I_{T}=\frac{I_{0}}{1+c^{*}\exp\left(-\frac{E_{a}}{kT}\right)}$$

Quantum efficiency is the second important factor to be considered in practical applications. The quantum efficiency of the phosphor doped with In^3+^ ions was tested, as exhibited in Figure 3d. Due to the limitation of the instrument, the test range was between 400 and 850 nm, and the internal quantum efficiency of its S1 part was measured as 15.80%. Considering the spectrum of the S2 part, the actual quantum efficiency was calculated as 42.21%, which is 34% higher than that of the quantum efficiency when undoped with In^3+^ ions (IQE = 31.60%). Its external quantum efficiency is correspondingly increased to 21.94%. It can be seen that the doping of In^3+^ ions improved the multifaceted properties of SGGO:Cr^3+^.

### 2.4. Application Exploration

**Figure 4:**
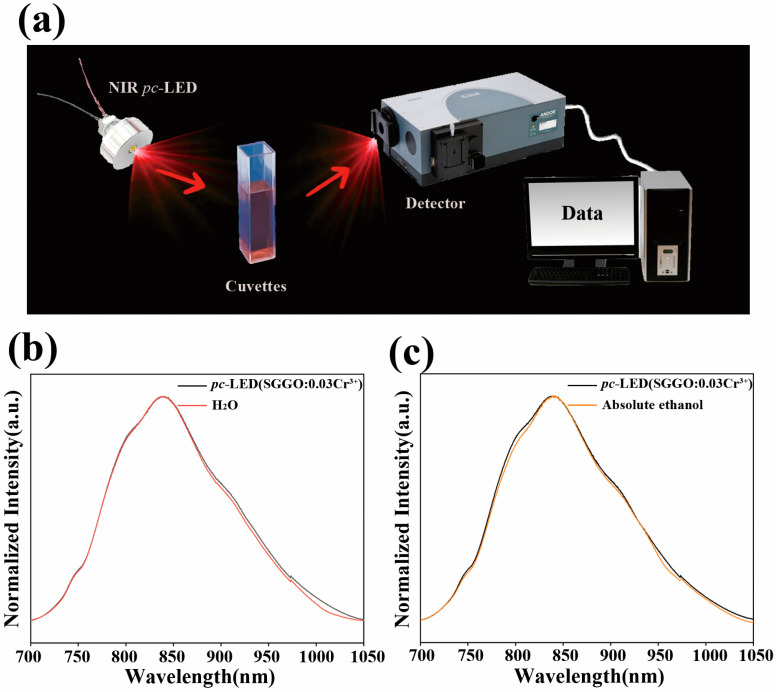
(**a**) Schematic diagram of the liquid detection device; comparison of the spectra of the *pc*-LED after passing through (**b**) water and (**c**) anhydrous ethanol.

The main component of anhydrous ethanol is C_2_H_6_O, which mainly absorbs light in the band near 800 nm; the main component of water is H_2_O, which mainly absorbs light in the band near 900 nm. As shown in the schematic diagram in Figure 4a, a 430 nm blue InGaN chip with SG_1.98_I_0.02_GO:0.03Cr^3+^ is encapsulated in a pc-LED, and the prepared pc-LED is used as a light source to irradiate the side of the cuvette. Due to the different absorption wavelengths of the different groups of light, there is a corresponding lack of wavelengths in the light transmitted through the cuvette. The spectrometer on the other side can output the corresponding data for comparison with the standard spectrum so as to achieve the purpose of non-destructive testing. Figure 4b,c exhibit comparisons of the chip light with the standard spectrum after passing through water and anhydrous ethanol, respectively. It can be seen that water has obvious absorption at 900 nm, and anhydrous ethanol has obvious absorption at 800 nm, so different liquids can be recognized using different absorption ranges, which proves that the *pc*-LED has good application prospects in non-destructive testing.

## 3. Materials and Methods

### 3.1. Sample Preparation

A series of Sr_3_Ga_2−2*x*_In*_x_*Ge_4_O_14_:0.03Cr^3+^ (*x* = 0, 0.2, 0.4, 0.8, 1.2, 1.6, 2.0) phosphors were successfully synthesized via the high-temperature solid-phase reaction method. Gd_2_O_3_ (99.9%, Aladdin, Riverside, CA, USA), ZnO (99%, Aladdin, USA), Ga_2_O_3_ (99.999%, Zhu Zhou Heng Ma, Zhuzhou, China), GeO_2_ (99.9999%, Zhu Zhou Heng Ma, China), Cr_2_O_3_ (99%, Kermel, Tianjin, China), and 3%H_3_BO_3_ (99.5%, Aladdin, USA) as a flux were applied as raw materials. The raw materials were weighed according to the stoichiometric ratio, and then all the weighed drugs were poured into an agate mortar and evenly ground for more than 30 min to make a homogeneous mixture. Then, the resulting powder was placed into a corundum crucible, whose main component was Al_2_O_3_, which was labeled with a good order, and then put into a high-temperature furnace. The temperature was raised at a rate of 5 K/min, starting from 273 K to 773 K, and held at a temperature for 30 min, and then the temperature was increased to 1173 K, and held at that temperature for 360 min, to complete the first sintering. After waiting for its natural reduction to room temperature, it was removed and ground for 30 min. Then, the second sintering was carried out by increasing the temperature at a rate of 5 K/min, starting from 273 K to 773 K, holding for 30 min, and then increasing to 1423 K, holding for 360 min to complete the sintering. Finally, the sintered samples were milled again to make a uniform and fine particle powder for subsequent characterization and testing.

### 3.2. Preparation of pc-LED

A certain amount of phosphor in proportion to the organic silica gel was mixed and stirred for more than 30 min. Afterwards, the mixed sample entered the defoaming process (air bubbles lead to chip light leakage). Mixed and defoamed samples were placed on the blue LED chip and then sent through the LED test system to determine device-related performance. Once the device met the necessary requirements, it was placed in the drying oven at 423 K for one hour. Afterwards, the device was cooled to room temperature for the next step of the experiment.

### 3.3. Sample Characterization

Measurements were carried out using an X-ray diffractometer (Bruker D8 Advance, München, Germany) at 40 kV and 40 mA and Cu-Ka (l = 1.54056 Å) irradiation in the 2θ range from 10° to 80° in 0.02° scanning steps. General Structure Analysis System (GSAS) software (version 1251) was used to optimize and analyze the structure. The morphology of the powder samples was investigated using a field-emission scanning electron microscope (FEI Nova Nano SEM 450, Peabody, MA, USA), and the elemental mapping results were obtained via energy-dispersive spectrometry (EDS) on the SEM equipment. Elemental composition was measured using energy dispersive spectroscopy and an X-ray spectrometer connected to the SEM. Fluorescence spectra of SGGO were tested using a transient steady-state fluorescence spectrometer (HORIBA FLuorolog-3, Kyoto, Japan) equipped with a variable-temperature liquid helium optical thermostat. Temperature emission spectra were measured from room temperature to 423 K in 283 K intervals using an external heater. The excitation wavelength was 431 nm. Measurements of electroluminescence properties were performed with a spectrometer (HORIBA FLuorolog-3, Kyoto, Japan) with an external DC power supply of 3 V and a current of 100 to 400 mA.

## 4. Conclusions

Based on the study of the luminescence properties of SGGO:0.03Cr^3+^, SGGO:0.03Cr^3+^, which has a wider half-peak width and a luminescence position more inclined to the long-wave direction, was selected as the object for further modulation study on the premise that it is more conducive to practical applications. Based on the cation substitution strategy, a series of Sr_3_Ga_2−2*x*_In*_x_*Ge_4_O_14_:0.03Cr^3+^ (*x* = 0–0.05) near-infrared phosphors with wider half-peaks and stronger luminescence intensity were obtained by replacing Ga^3+^ ions with In^3+^ ions in the matrix at a certain concentration. The large-radius In^3+^ replaced Ga^3+^ ions affecting the crystal field strength, leading to spectral red shift, which was explained in detail by analyzing the electron cloud expansion effect, crystal field strength changes, and bit pattern coordinate diagram. In addition, the temperature stability and quantum efficiency after the modulation were significantly improved, which are more favorable for practical applications. Finally, the phosphor Sr_3_Ga_1.98_In_0.02_Ge_4_O_14_:0.03Cr^3+^ with the strongest emission intensity was selected to be encapsulated into a *pc*-LED, which was applied in NDT experiments to successfully realize the recognition of water and anhydrous ethanol, proving its application potential in the field of NDT.

## Data Availability

Data are contained within the article and Supplementary Materials.

## Supplementary Materials

The following supporting information can be downloaded at: https://www.mdpi.com/article/10.3390/molecules28248059/s1, Figure S1: (a) Refined XRD pattern of SGGO:0.03Cr^3+^; (b) refined XRD pattern of SG_1.98_I_0.02_GO:0.03Cr^3+^; (c) refined XRD pattern of SG_1.99_I_0.01_GO:0.03Cr^3+^; (d) refined XRD pattern of SG_1.97_I_0.03_GO:0.03Cr^3+^; (e) refined XRD pattern of SG_1.96_I_0.04_GO:0.03Cr^3+^; (f) refined XRD pattern of SG_1.95_I_0.05_GO:0.03Cr^3+^; Figure S2: Three-dimensional temperature spectra of SG_1.98_I_0.02_GO:0.03Cr^3+^.
