# Supplementary material for: Achieving Luminescence of Sr3Ga1.98In0.02Ge4O14:0.03Cr3+ via [In3+] Substitution [Ga3+] and Its Application to NIR pc-LED in Non-Destructive Testing
Source: Molecules. 2023 Dec 13;28(24):8059. doi: 10.3390/molecules28248059 (PMC10745490; doi:10.3390/molecules28248059)
Supplement: Supplementary file 1 [file molecules-28-08059-s001.zip › molecules-2737380-supplementary.pdf]

Supplementary materials

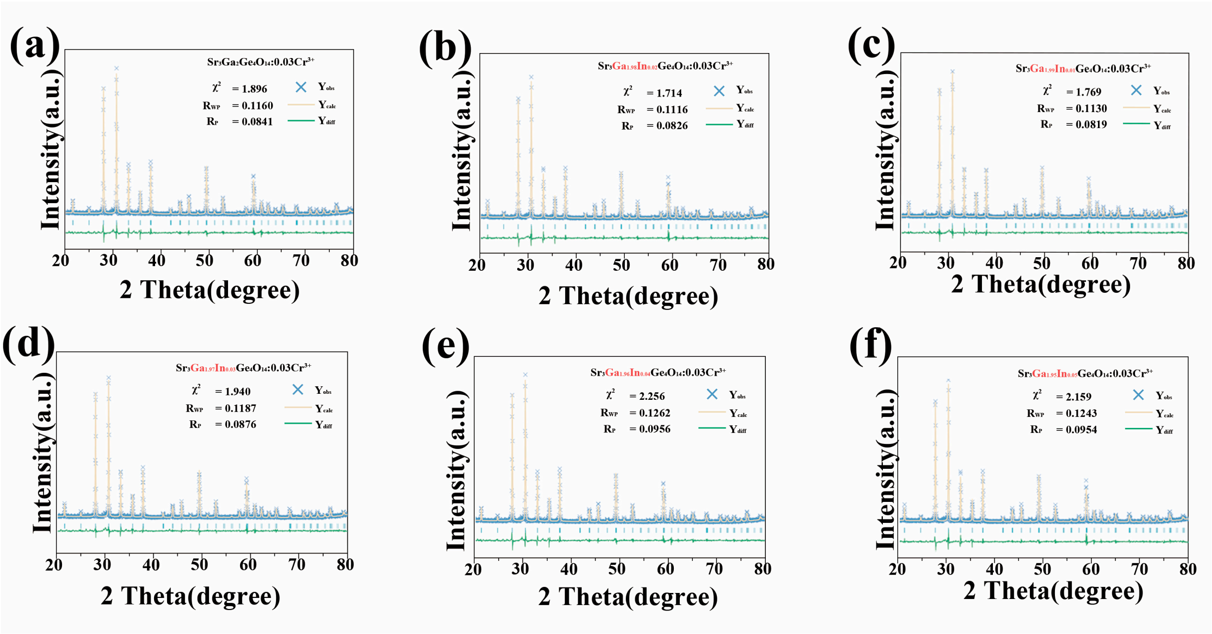

Figure S1. (**a**) Refined XRD pattern of SGGO:0.03Cr^{3+}; (**b**) Refined XRD pattern of SG$_{1.98}$I$_{0.02}$GO:0.03Cr^{3+}; (**c**) Refined XRD pattern of SG$_{1.99}$I$_{0.01}$GO:0.03Cr^{3+}; (**d**) Refined XRD pattern of SG$_{1.97}$I$_{0.03}$GO:0.03Cr^{3+}; (**e**) Refined XRD pattern of SG$_{1.96}$I$_{0.04}$GO:0.03Cr^{3+}; (**f**)refined XRD pattern of SG$_{1.95}$I$_{0.05}$GO:0.03Cr^{3+}.

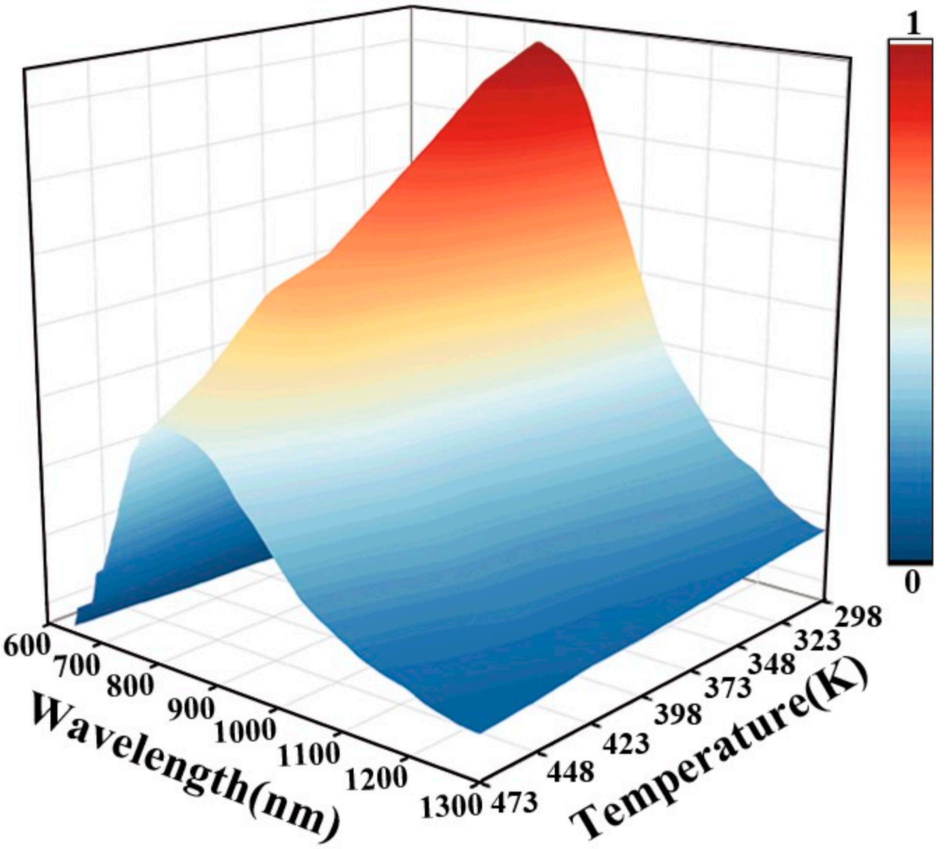

Figure S2. Three-dimensional temperature spectra of SG$_{1.98}$I$_{0.02}$GO:0.03Cr^{3+}
